# Editorial: Neurodegeneration: From Genetics to Molecules (Part II)

**DOI:** 10.3389/fncel.2021.794630

**Published:** 2021-11-17

**Authors:** Karla Guadalupe Carvajal, Rocío Martínez De Pablos, Victoria Campos-Peña

**Affiliations:** ^1^Laboratorio de Nutrición Experimental, Instituto Nacional de Pediatría, Mexico City, Mexico; ^2^Department of Biochemistry and Molecular Biology, Faculty of Pharmacy, University of Seville, Seville, Spain; ^3^Laboratorio Experimental de Enfermedades Neurodegenerativas, Instituto Nacional de Neurología y Neurocirugía, Manuel Velasco Suárez, Mexico City, Mexico

**Keywords:** neurodegeneration, oxidative stress, reactive oxygen species, dementia, inflammation

Among non-transmissible chronic diseases, neurodegenerative complications have become a big challenge in public health. Elongation of human lifespan, due in part to better health services, lifestyle changes, and improvements in medicine and nutrition, has brought up in consequence, an emergence in growing scientific knowledge on fields related to aging and longevity physiology. This new social context faces human medicine to novel concerns that includes coping with chronic diseases that affect life quality at elderly and that increasingly, appear earlier in mid-age and young people.

This special issue offers a broad overview of current knowledge of the molecular and genetic mechanisms that underly neurodegenerative processes; present in various diseases of the central nervous system (CNS) such as Alzheimer's (AD), Parkinson's (PD), and temporal lobe epilepsy (TLE) among others. Here, we will address aspects such as the role of β amyloid (Aβ) and tau protein in neurodegeneration, vascular endothelial growth factor (VEGF) in neuroprotection processes, signaling pathways such as NGF/TrkA/Akt/Nrf2 and AMPK/mTor, protein trafficking, DNA repair, apoptosis, inflammation, excitotoxicity, metal exposure, oxidative stress, and nanotechnology.

*Neurodegeneration: From Genetics to Molecules II*, is a multidisciplinary Research Topic that aims to interest basic researchers from several fields of neuroscience research, to seek and suggest new routes for the management of these diseases and help the development of more effective therapeutic approaches. Based on this, we selected this collection of manuscripts with the purpose of presenting the most innovative advances that help to solve unanswered questions and with this, opening new scenarios focused on the development of innovative strategies for learning and improving the treatment of neurodegenerative diseases.

Repair mechanisms in the brain play an important role; in this respect, Cardenas-Rivera et al. evaluated the neuroprotective mechanisms mediated by VEGF in the acute phase of stroke. To do this, they used an *in vivo* model produced by the transitory occlusion of the middle cerebral artery in the rat; the i.c.v. of VEGF favored an increase in neuronal survival, as well as a decrease in infarct volume. These results are directly related to the preferential activation of VEGF receptor 1 (VEGFR1), which has a significant role in the modulation of the inflammatory response and the polarization of the microglia toward a protective phenotype; suggesting, that this receptor could be a target for the development of therapeutic approaches during the acute phase post-stroke (Cardenas-Rivera et al.).

Glutathione (GSH) is an intracellular antioxidant molecule, which participates in several functions, such as proliferation, cell differentiation, and apoptosis. It is known that during aging, as well as in neurodegenerative and neuropsychiatric diseases, an alteration in the cellular GSH pools, and a downregulation of GSH-dependent enzymes are present. The increase in circulating nerve growth factor (NGF), an activator of antioxidant pathways, triggers a protective response, which involves an increase in GSH levels in CNS. Valdovinos-Flores et al. showed that peripheral administration of L-buthionine-S-R-sulfoximine (BSO) increases peripheral NGF, which activates the NGF/TrkA/Akt pathway in striatal neurons and leads to a neuroprotective response. Activation of this pathway induces an increase in the expression of genes involved in the uptake of the aminoacids L-cys and L-cys2, as well as glutamate-cysteine ligase modifier (gclm) subunit, which are related to GSH synthesis and transport from the blood to the neuronal parenchyma. The results obtained by these authors are of great importance since they demonstrate that the peripheral reduction of GSH significantly increases the circulating NGF, favoring the neuroprotective response; allowing the development of new studies to elucidate the role that peripheral NGF has on the modulation of GSH homeostasis in the CNS, and opening new possibilities in the progress of therapeutic strategies for neurodegenerative diseases (Valdovinos-Flores et al.).

Qiu et al. conducted a review of TPM21 evaluating the role of this protein in the development of AD. TMP21 is a type I transmembrane protein, which belongs to the p24 family. It is expressed in tissues such as the brain, heart, liver, lung, pancreas, etc. It has a significant role in protein trafficking and maturation, so it plays a critical role in maintaining physiological functions. The authors point out that the dysregulation of TMP21 is involved in the pathogenesis of AD. First, they indicate the regulation of TMP21 expression, whose gene is located on chromosome 14q24.3 and includes five exons and four introns, is positively regulated by nuclear factor of activated T-cells (NFAT), as well as another transcriptional factors such as CREB, AP1, and YY1F. The authors noted that TMP21 regulates AβPP trafficking, affecting Aβ production, thus, dysregulation of TMP21 promotes Aβ generation by modulating APP trafficking/stability and regulating the processing of APP by γ-secretase activity, while it potentially regulates the expression and activity of BACE1. Finally, they mention that TMP21 dysregulation could promote tau phosphorylation and neuronal apoptosis, contributing to synaptic impairment and neuronal loss; therefore, modulating the expression of TMP21 could be a potential therapeutic target for the treatment of AD (Qiu et al.).

In recent years it has been documented that the CNS is exposed to constant oxidative damage, due to the presence of reactive oxygen species (ROS), which causes damage to the neuronal genome including double-stranded DNA breakage (DSBs). This DNA damage is not only related to the aging process, but also to neurodegenerative diseases. Guo et al. evaluated the role of RAD6 in double-stranded DNA repair, using RAD6-deficient mice and hippocampal cultures to which a siRNA against RAD6B was introduced. They compared RAD6-deficient mice with wild-type mice after DNA damage induced by X-irradiation. The results obtained demonstrated that RAD6B-deficient mice exhibit deficits in learning and memory processes. It was also observed that RAD6 is essential for neuronal DNA damage response (DDR), since its deficiency causes defects in DSBs repair, generating genomic instability that leads to neurodegeneration (Guo et al.).

Yao et al. evaluated the phagocytic and anti-inflammatory functions dependent on TREM2 in a non-immune and non-phagocytic cell model, which does not express TREM2 or DAP12. To do this, they stably co-transfected HEK293 cells with TREM2 and DAP12 and evaluated the effects of some TREM2 mutations associated with AD. The results demonstrated that HEK293 cells had the ability to engulf bioparticles of *Escherichia coli*, only when they co-express TREM2 and DAP12, and that the presence of mutations in TREM2 significantly decreases the phagocytic process. In the same way, co-expression of TREM2 and DAP12 prevented NFκB activation in response to PMA treatment, and the TREM2 R47H mutation prevented PMA-induced TREM2-dependent inhibition of NFκB. Finally, TREM2-dependent phagocytosis requires the activation of SYK/PI3K/AKT/PLCγ pathways, demonstrating that phagocytic and anti-inflammatory activities are mediated by different signaling pathways (Yao et al.).

Chronic inflammation is a feature present in many neurodegenerative diseases, such as, AD. The inflammatory process is controlled and culminated through negative feedback mechanisms that allow the restoration of the organism's homeostasis. When this does not occur, a detrimental process is generated that can lead to neurodegeneration. Inflammation resolution is an active process that involves not only the decrease in the proliferation and maturation of immune cells or the inhibition of the secretion of inflammatory mediators, as it also involves the repair of damaged tissues. This process is conducted by a group of specialized pro-resolving lipid mediators (SPMs) such as maresins (MaR1). Yin et al. investigated the effects of MaR1 on pathological changes and behavioral deficits in a mouse model of AD by Aβ42 microinjection. Their results showed that treatment with MaR1 decreased the activation of astrocytes and microglia, by decreasing the production of pro-inflammatory cytokines, induced by the microinjection of Aβ42. In the same way, it reduced the apoptosis pathways, and MaR1 significantly improved cognitive impairment and enhanced cell survival, suggesting that inflammation resolution may be a potential therapeutic target for AD (Yin et al.).

In recent years, new functions have been identified for caspases that go beyond being considered solely as regulators of apoptosis or inflammation. Espinosa-Oliva et al. conducted a mini-review focused on the “non-apoptotic” functions of caspases in the CNS. In this work, the participation of caspases in the activation of inflammasome, pyroptosis, and necroptosis in neuronal cells is discussed. The function of caspases in the synapse and the processing of aggregates, present in various neurodegenerative diseases, is also evaluated. These types of functions, other than apoptosis, may be related to the sequestration of effector caspases in different subcellular compartments and to the processing of substrates that are not related to cell death. An important example is procaspase-3, which can mediate mitochondrial biogenesis, regardless of its enzymatic function. Caspase-3 also regulates embryonic stem cells (ESCs) differentiation through Nanog processing. Finally, they point out that the functions observed in caspase-3 can be investigated for other caspases, opening new lines of research to elucidate these functions beyond apoptosis and to evaluate them in other types of cells, different from those of the CNS (Espinosa-Oliva et al.).

Microglia, the immune cells of the CNS, play a fundamental role in brain homeostasis and in the neuroinflammation process. Liu et al. reviewed the role of microglia in the development of PD. They discuss that microglia have pro-inflammatory and anti-inflammatory activities, which depend on the stage and severity of the disease. Microglial polarization is a dynamic process that involves the release of inflammatory mediators such as cytokines, chemokines, ROS, growth factors, reactive nitrogen species (RNS), and prostaglandins (PG). In PD, damaged dopaminergic neurons are capable of causing neuroinflammation, oxidative stress, and cytokine-receptor-mediated apoptosis; this favors peripheral leukocyte recruitment, generating a feedback mechanism that results in exacerbation of the neurodegenerative process. Likewise, they indicate the interaction of microglia with other cell types such as neurons, astrocytes, mast cells, the microbiome-intestine-brain axis as well as α-Synuclein-microglia interaction, which have a significant role in the regulation of microglial activation and neuroinflammation. Finally, they point out that since the activation of the microglia is a key point to understand PD, the therapeutic strategy should be focused at restoring the immune balance, instead of inhibiting the immune response (Liu et al.).

In the same way, Cheray et al. evaluated the role and functionality of microglia in primary brain tumors and AD. They reviewed several reports in the literature pointing out that there is an inverse relationship between AD and the appearance and development of glioblastoma mediated by microglia. The microglia triggers the activation of signaling cascades such as AKT-mTOR, PI3K, and PAM, that favor the tumor microenvironment, which are not present in AD. In AD, microglia is found around the amyloid plaques, while in cancer they constitute the tumor mass as well as support the expansion and invasion of glial cells into the tumor. The manuscript suggests that the key point that allows this inverse correlation is also found in the expression of specific proteins, the activation of some genes, and signaling pathways (Cheray et al.).

Metaxas et al. proposed new biomarkers for identifying early stages of AD progression, such as the synaptic vesicle glycoprotein 2A (SV2A), as a measure of neuronal density, inflammatory markers, and tau phosphorylation by using modern neuroimaging techniques. They found a positive correlation of these three biomarkers with the damage of the neuronal tissue, mainly in the frontal gyrus, a region that is vulnerable to Aβ deposition, which could provide new approaches to early diagnosis of AD dementia. Although a small pilot study, the released results give new insights in the use of novel imaging technologies that could serve as tools in clinical diagnosis for opportune intervention and treatment of brain diseases (Metaxas et al.).

On the other hand, Silva-Adaya et al. showed in an integrative work, the role of environment metal pollution in the neurodegenerative processes, pointing out its importance on the growing incidence of chronic brain diseases such as AD. This work demonstrated that exposure to inorganic arsenic in healthy mice importantly affects the antioxidant capacity and amino acids transporter gene expression of brain tissues, which in turn, may predispose the organ to further damage or accelerate neurodegenerative pre-existing processes. These findings are valuable clues in preventing elderly complications, either by an opportune diagnosis, or by improving preventive therapies based on boosting the antioxidant capacity by nutritional support and healthier lifestyle. But, moreover, this work alerts about the imperative necessity of taking care of nature milieus, including food and tap water, from toxic pollutants derived from human activity, in order to preserve a healthful future in a context where longer lifespan has become a novel health challenge (Silva-Adaya et al.).

Concerning neurodegenerative chronic diseases, a novel proposal of treatment is presented by Saliba et al.. Here, they show the importance of cannabinoid receptors (CR) in the control of neuroinflammation and exocitoxicity by using N-arachidonoylphenolamine (AM404) as a CR agonist. They also revealed the underlying mechanisms triggered by AM404, glutamate and calcium release that activate in turn the G-protein-related pathways that lead to cell death, and constitute one of the main causes of neurodegeration in brain regions such as hippocampus and hypothalamus, often associated to mental and neuronal diseases such as AD, PD, and Huntington. They also found that modulation of CRs might reduce the pathways that conduce to inflammatory processes, by inhibiting the release of pro-inflammatory cytokine L-1β from microglia cells. Thus, they open new alternatives by targeting inflammation and neurotoxicity using CRs agonists, not only AM404, but many other, new and old molecules, that interact with these proteins (Saliba et al.).

Regarding pioneering strategies for diagnosis and therapy, Luo et al. in their review, present a detailed description of modern methods for the diagnosis of chronic CNS conditions, such as AD, PD, and amyotrophic lateral sclerosis (ALS), and propose a novel tool based on the use of iron oxide nanoparticles (IONPs), serving as highly sensitive drug carriers and contrast agent that reach specific brain regions affected in these diseases. They point out the advantage of IONPs vs. orthodox invasive techniques, such as biopsying and autopsy diagnosis, which present low sensitivity and are useful only during the late onset of the diseases, besides the little patient acceptance. By contrast, IONPs represent an innovative contrast agent tool that allows detections at early stages of neurodegenerative hallmarks by non-invasive imaging, thanks to their capacity to cross the blood–brain barrier (BBB) and relatively easy clearance, with a low grade of toxicity (Luo et al.).

More insights in the mechanisms involved in the neurodegenerative processes that accompany AD and epilepsy are revised in the work of Toral-Rios et al.. Here, they underline the role of the signaling pathway of glycogen synthase kinase 3β (GSK3β) in leading the phosphorylation of key proteins such as tau, the N-methyl-D-aspartate receptor (NMDAR), and pro-apototic molecules Bax and Bcl, which promote structural changes and organelle dysfunction that turn them into toxic components for the cell, such as neurofibrillary tangles (NFTs), excitotoxicity by the overstimulation of NMDAR and mitochondrial dysfunction. Detailed mechanisms of how GSK3β may interact with these pivotal proteins are discussed. The participation of upper signaling cascades, such as PI3K/Akt/mTor and Wnt/β-catenin, which are disrupted in some chronic metabolic conditions, may be involved in the overstimulation of GSK3β. These findings remark the importance of GSK3β modulation to maintain cellular homeostasis in neurodegenerative diseases, so that the proposal of this protein as a therapeutic target is open, in order to offer better alternatives for patients suffering from brain declining function. Besides, deeper studies about the nature of post-translational modification (PTM) of key proteins are encouraged, since, as pointed by the authors, they may play a significant role in modulating the function of the entire cell and thus, of the whole organ (Toral-Rios et al.).

In the same way, Han et al. extensively described the steps involved in aggregation of α-Synuclein, and the effect on molecular components involved in the formation of toxic fibrils that damage neurons and lead to the progressive motor impairment in PD. Their discussion focuses on the local domains of the protein that mediate its aggregation, the physic-chemical determinants that undertake the phase separation and that finally lead to aggregation and fibrils formation. Cellular oxidative stress is commonly associated with chronic CNS diseases, standing out as the primary involved mechanism. Under this context, PTM such as phosphorylation, SUMOylation, nitration, and O-gcNacylation in α-Synuclein have been revealed as a critical step in the transition of the protein from a monomeric to an oligomeric toxic state. Also, interaction of α-Synuclein with other cellular components such as mitochondria, lipid membranes, inflammatory cells, and genetic mutations are reviewed, warning the complex network of factors surrounding the role of this protein and its conversion into a toxic agent for PD, either by modifying its structure into an aggregating form, or by mediating signal cascades that evoke cell death (Han et al.).

Auchter et al. presented an alternative use of methylene blue (MB) as neuro-protective agent, facing diminished vascular perfusion that often precedes cerebral hypoperfusion in neurodegenerative processes. By using a model of permanent bilateral carotid artery occlusion (two-vessel occlusion; 2VO), Auchter et al. underlie the mechanistic effects of oxygen deprivation and their contribution to brain cellular damage. They monitor cytochrome c oxidase activity (CO) as a marker of mitochondrial electron chain function, over different regions of the brain, and demonstrated that low doses of MB may somewhat replace electron supply into the oxidative phosphorylation to maintain ATP production under brain ischemia and thus, preventing neurodegeneration and memory impairment. Therefore, regional CO activity impairment occurs in response to hypoperfusion, causing specific injury of clue brain regions that compromise cognition. However, electron donors such as MB may serve to reduce the extent of damage that could trigger neurodegeneration (Auchter et al.).

In the last review, Luna-Viramontes et al. revised the role of tau protein, the second element involved in tissue damage during AD. Tau protein belongs to the family of microtubule-associated proteins and undergoes PTMs that change its properties, such as hyperphosphorylation and cleavage, which render it less soluble and cause aggregation that forms fibrillar structures known as NFTs. The molecular mechanisms underlying the critical step on NFTs formation are the foundation of the minimal paired helical filaments, which serve as core for the growth of the NFTs. This mechanism takes place early during AD development, even before clinical symptoms appear. An important issue in NFTs formation are the signal pathways involved, mainly kinases such as GSK-3β, cell division protein kinase 5 (CDK5), AMP-activated protein kinase (AMPK), and protein kinase A, which are often affected by metabolic disturbances. Thus, the implication of preserving energy metabolism is relevant in the prevention of neurodegenerative processes. In brief, incipient steps in NFT formation appear to be a key control point in detection, prevention, and treatment of the AD and other related pathologies (Luna-Viramontes et al.).

Taken together, all the papers in this issue, present new insights on the cellular mechanisms that lead the subtle neurodegenerative progression behind chronic diseases such as AD, PD, Huntington, ALS, and epilepsy. They also reveal new biomarkers and protein targets aimed to help on early diagnosis, development of potential more effective drugs, and even preventive therapies that guarantee a higher quality of life. This is an issue especially important in a social context where longer life expectancy emerges along with new challenges imposed by the need of deep knowledge of CNS molecular physiology.

They also emphasize the importance of basic research that reveals at a molecular detail, the components and interactions among genes, external milieu, and lifestyle, that raise the perfect puzzle to damage or to heal the brain cells that control function in the aging brain, required to reduce the disability on sustaining longer lives from the future population.

## Author Contributions

VC-P and KC prepared the editorial. RMP reviewed and edited the manuscript. VC-P, KC, and RMP contributed significantly to the review and editing of each manuscript accepted on this topic.

## Conflict of Interest

The authors declare that the research was conducted in the absence of any commercial or financial relationships that could be construed as a potential conflict of interest.

## Publisher's Note

All claims expressed in this article are solely those of the authors and do not necessarily represent those of their affiliated organizations, or those of the publisher, the editors and the reviewers. Any product that may be evaluated in this article, or claim that may be made by its manufacturer, is not guaranteed or endorsed by the publisher.

